# Docosahexaenoic acid regulates vascular endothelial cell function and prevents cardiovascular disease

**DOI:** 10.1186/s12944-017-0514-6

**Published:** 2017-06-15

**Authors:** Kazuo Yamagata

**Affiliations:** Department of Food Bioscience and Biotechnology, College of Bioresourse, Science, Nihon University (NUBS), 1866, Kameino, Fujisawa, Kanagawa 252-8510 Japan

**Keywords:** DHA, Endothelial cells, Cardiovascular disease

## Abstract

Docosahexaenoic acid (DHA) is present in high concentrations in salmon, herring, and trout. Epidemiologic studies have shown that high dietary consumption of these and other oily fish is associated with reduced rates of myocardial infarction, atherosclerosis, and other ischemic pathologies. Atherosclerosis is induced by inflammation and can lead to acute cardiovascular events and extensive plaque. DHA inhibits the development of inflammation in endothelial cells, alters the function and regulation of vascular biomarkers, and reduces cardiovascular risk. It also affects vascular relaxation and constriction by controlling nitric oxide and endothelin 1 production in endothelial cells. DHA also contributes to the prevention of arteriosclerosis by regulating the expression of oxidized low density lipoprotein receptor 1, plasminogen activator inhibitor 1, thromboxane A2 receptor, and adhesion molecules such as vascular cell adhesion molecule-1, monocyte chemoattractant protein-1, and intercellular adhesion molecule 1 in endothelial cells. Recent research showed that DHA reduces the increase in adhesion factor expression induced by lipopolysaccharide by suppressing toll-like receptor 4. A new mechanism of action of DHA has been described that is mediated through endothelial free fatty acid receptor 4, associated with heme oxygenase 1 induction by Nrf2. However, the efficacy and mechanisms of action of DHA in cardiovascular disease prevention are not yet completely understood. The aim of this paper was to review the effects of DHA on vascular endothelial cells and recent findings on their potential for the prevention of circulatory diseases.

## Background

That a relation exists between cardiovascular disease (CVD) and omega-3 fatty acids (n-3 FA) first emerged in 1976 when Dyerberg et al. discovered that the Inuit of Greenland had a remarkably low incidence of CVD compared with the population of Denmark. A large difference in the consumption of eicosapentaenoic acid (EPA) and docosahexaenoic acid (DHA) was considered to be the principal underlying factor [1–4]. These early studies led to epidemiologic studies of cardiovascular disease prevention with n-3 FAs. However, some authors have questioned this conclusion. The main criticisms related to the systems used for health monitoring and for registration of the cause of death in Greenland [5, 6]. Nevertheless, many epidemiologic and clinical studies have provided evidence that the polyunsaturated n-3 FA DHA in fish and fish oils provides cardiovascular disease protection. In several prospective cohort studies, the benefit ascribed to n-3 FA differed. The results were divided between those that were effective [7–10] and those that were not effective [11, 12]. Consequently, a large-scale meta-analysis of cohort studies was performed to examine the association between fish consumption and coronary heart disease mortality [13]. Based on 11 eligible studies and 13 cohorts (222,364 persons with an average 11.8 years of follow-up), fish intake was found to be inversely associated with the hazard ratio for coronary heart disease mortality. An increase in fish intake of 20 g/day was associated with a 7% decrease in the risk of coronary heart disease mortality. Recently, in regard to the protective effect of n3-LCPUFA against CVD, a literature search was performed using PubMed and Medline entries between January 1, 2007 and August 31, 2013. Higher fish intake was associated with decreases in sudden cardiac death, stroke, myocardial infarction, and heart failure. These results supported the efficacy of 3 n-LCPUFA in hypertriglyceridemia and heart failure [14]. The results indicated efficacy of 3 n-LCPUFA for hypertriglyceridemia [14] and heart failure [15]. Alpha-linolenic acid (ALA) is a substrate for the synthesis of EPA and DHA, but the conversion rates are low [16, 17]. Accordingly, the DHA intake in food is important.

As DHA is present in deep water fish such as salmon, mackerel, clupea, sardine, and tuna [18, 19], it was suggested that the effect was attributable to variations in the intake of DHA. The fish lipids derived from these diets prevent coronary artery disease, heart failure, and cardiac arrhythmias, for example, and lower the mortality rate from these CVDs [20, 21]. In addition, it has been found that the circulating concentrations of DHA and EPA were inversely related to CVD incidence [22].

It has been shown that several mechanisms account for the protective effects of EPA or/and DHA against CVD. For example, n-3 FAs in fish oils inhibit cardiac arrhythmias and decrease plasma triglycerides, but have little effect on low-density (LDL) or high-density (HDL) lipoprotein cholesterol levels [23]. n-3 FA may inhibit inflammation [24], and may prevent atherosclerosis and plaque rupture by an anti-inflammatory effect [25]. In older and hypertensive subjects, a high intake of fish oil may lower blood pressure [26]. In patients with Type 2 diabetes, moderate oral supplementation with DHA improved platelet function and oxidative stress [27]. The relations between polyunsaturated fatty acids (PUFAs) and CVD were investigated in 2837 US adults, and DHA was found to be inversely associated with CVD incidence [24]. DHA may have anti-thrombotic, anti-atherogenic, anti-arrhythmic, and vasoprotective effects [28]. With regard to anti-thrombotic activity, DHA decreases platelet aggregation and blood viscosity and also affects clotting factors. In relation to anti-atherogenic activity, it reduces plasma triglycerides, inhibits the migration and growth of vascular smooth muscle cells (SMC), and inhibits the production of cytokines and adhesion molecules. The anti-arrhythmic activity results from effects on membrane ion channels, increases in VF threshold (HRV), alterations in membrane fluidity, and limitation of ischemic damage. Furthermore, it has been shown that DHA induces the expression of nitric oxide synthase (eNOS) and stimulates the production of nitric oxide in vascular endothelial cells [29]. It also prevents vascular inflammation and decreases monocyte adhesion induced by oxidized LDL (ox-LDL) in coronary endothelial cells [30].

It has been shown that DHA reduces endothelial cell injury, decreases an inflammatory marker, and prevents CVD. In particular, lectin-like oxidized LDL receptor-1 (LOX-1) may contribute to vascular endothelial dysfunction and enhance atherosclerosis [31, 32]. However, DHA inhibited TNF-α-induced gene expression related to endothelial dysfunction, including LOX-1 [33]. Furthermore, DHA inhibits induction of inflammation by controlling cell signal transduction and altering gene expression in the cells. It can inhibit tumor necrosis factor-alpha (TNFα)-induced vascular cell adhesion molecule-1 (VCAM-1) expression by reducing the NF-κB signaling pathway [34]. Its vasoprotective effects indicate that it improves vascular endothelial cell function, modulates receptor-agonist interactions, reduces blood pressure, and reduces end-organ damage. These properties of DHA help to prevent circulatory disease and to regulate many metabolic functions. Free fatty acid receptor 4 (FFAR4) is activated by n-3 PUFAs such as ALA, EPA, and DHA [35]. With regard to the mode of action of DHA, FFAR4 is likely to be related to the function of DHA [36, 38]. However, neither the efficacy nor the mechanism of action of DHA in CVD prevention is completely understood. Endothelial cell-dependent contractions are reduced when the release of nitric oxide is impaired by oxidative stress, ageing, hypertension, and diabetes [39]. The aim of this paper was to review the effects of DHA on vascular endothelial cells and recent findings on their potential for the prevention of circulatory diseases.

## Dietary sources of DHA and its biosynthesis from alpha-linolenic acid

**Fig. 1:**
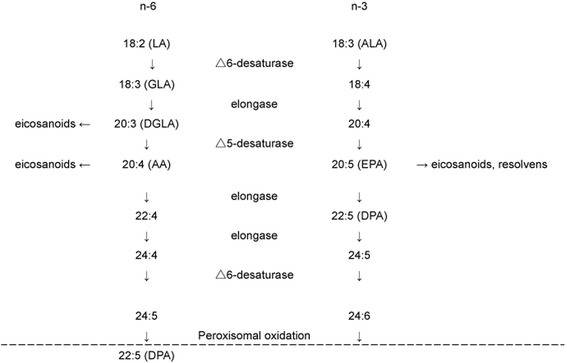
Enzymatic conversion of LA to longer-chain n–6 PUFAs and ALA to longer-chain n–3 PUFAs. The conversion of ALA to EPA and DHA is catalyzed by elongase and a desaturase enzyme. The desaturase shares ALA (18:3n-6) with elongase and the conversion ratio is affected by the quantity of substrate. As such, when n-6 PUFA levels are high, the amount that is converted to n-3 PUFA decreases. On the other hand, EPA can be converted to DHA or eicosanoids. *Abbreviations:* AA, arachidonic acid; ALA, Alpha-linolenic acid; DPA, docosapentaenoic acid; DGLA, dihomo-γ-linolenic acid; EPA, eicosapentaenoic acid; GLA, gamma-Linolenic acid; LA, Linolenic acid; PUFAs, polyunsaturated fatty acids

DHA is an n-3 FA component of the phospholipids of animal cell membrane, including those of the skin and retina, and is present in high concentrations in human brain tissue. It can be synthesized from alpha-linolenic acid (ALA) in vivo, and is supplied directly to infants in maternal milk. ALA is a substrate for the synthesis of the long-chain fatty acids EPA and DHA, but the conversion rates are low [40, 41], with only 0.2% being converted to EPA and 0.05% to DHA [41]. Therefore, the DHA consumed in food is important. The conversion of ALA to EPA and DHA is catalyzed by elongase and a desaturase enzyme [40] (Fig. 1). The desaturase shares ALA with elongase and the conversion ratio is affected by the quantity of substrate. When n-6 PUFA is abundant, the amount converted to n-3 PUFA decreases. On the other hand, EPA can be converted to DHA or eicosanoids. Many eicosanoids induce inflammation. But there is much greater potential for inflammatory activity with n-6 than with n-3 PUFAs, which are less inflammatory [41]. For example, prostaglandins (PGs), thromboxanes (TXs), and leukotrienes are representative eicosanoids that enhance inflammation. Also, competition between n-6 and n-3 FAs for metabolic enzymes influences the concentrations of various eicosanoids, which might contribute to the prevention of CVD by PUFAs [42]. Table 1 shows the main dietary sources of EPA, docosapentaenoic acid (DPA), and DHA [43]. Generally, deep water fish such as tuna, salmon, mackerel, herring, and sardines have high contents of EPA and DHA. In particular, salmon, trout and herring contain the highest concentrations of DHA [19].

**Table 1:** Major dietary sources of DHA^a^

							(mg/100 g)
	EPA	DPA	DHA		EPA	DPA	DHA
Anchovy	763	41	1292	Tuna, light (skipjack)	91	17	237
Herring, Atlantic	909	71	1105	Snapper	48	22	273
Salmon, farmed	862	393	1104	Flounder and sole	168	34	132
Salmon wild	411	368	1429	Clams	138	104	146
Mackerel, Atlantic	504	106	699	Grouper	35	17	213
Bluefish	323	79	665	Halibut	80	20	155
Sardines, Atlantic	473	0	509	Lobster	117	6	78
Trout	259	235	677	Scallops	72	5	104
Golden bass (tilefish)	172	143	733	Blue crab	101	9	67
Swordfish	127	168	772	Cod, PacifIc	42	5	118
Tuna, white (albacore)	233	18	629	Shrimp	50	5	52
Mussels	276	44	506	Catfish, farmed	20	18	69
Striped bass	169	0	585	Eggs	0	7	58
Shark	258	89	431	Chicken breast	10	10	20
Pollock, Atlantic	91	28	451	Beef	2	4	1
Oysters, wild	274	16	210	Pork	0	10	2
King mackerel	174	22	227				

*Abbreviations*: *DHA* docosahexaenoic acid, *EPA* eicosapentaenoic acis, *DPA* docosapentaenoic acid

^a^ Adapted from reference [43] Mozaffarian and Wu, 2012

## Protective effects of DHA on vascular endothelial dysfunction in vitro

### Vascular endothelial dysfunction and cardiovascular disease

Vascular endothelium consists of a single layer of cells lying between the blood and smooth muscle cells of blood vessel walls, where they play an important role in the homeostasis of vascular function. Vascular endothelial cells control blood pressure (BP), vascular permeability, blood coagulation, arterial stiffness, and inflammation. In addition, endothelial cells have many receptors, binding proteins, and transporters that regulate cell growth, apoptosis, and cell migration. The health of endothelial cells, which is essential for normal vascular function, is maintained by the synthesis of several molecules, including nitric oxide (NO), PGI2, and angiotensin II (AII) in response to physical and chemical stimuli [44–46]. For example, endothelial cells produce AII by the action of angiotensin converting enzyme (ACE), which is located in the cell membrane. The AII then stimulates the contraction of vascular smooth muscle cells to increase BP. Endothelial cells additionally release endothelin-1 (ET-1), which also causes smooth muscle to contract. Endothelial cells also regulate the production of NO, endothelium-derived hyperpolarizing factor (EDHF), and eicosanoids. The LOX-1 is a scavenger receptor that mediates the incorporation of ox-LDL into vascular cells. LOX-1 expression is recognized in atherosclerotic lesions and may contribute to vascular endothelial cell dysfunction and be an important risk factor for atherosclerosis [31, 32]. On the other hand, in certain diseases endothelial cells have the potential to increase the production of free radicals and thereby to promote vascular disorders. The balance between constriction and relaxation breaks down when the integrity and function of vessels are disturbed. Such dysfunctions can be caused by hypercholesterolemia, hypertension, diabetes, and smoking. Consequently, defective endothelial cell function is associated with a high risk of cardiovascular events [47] and leads to CVDs such as hypertension [48], atherosclerosis [49], and stroke [50].

### Mechanisms by which DHA prevents endothelial dysfunction

**Fig. 2:**
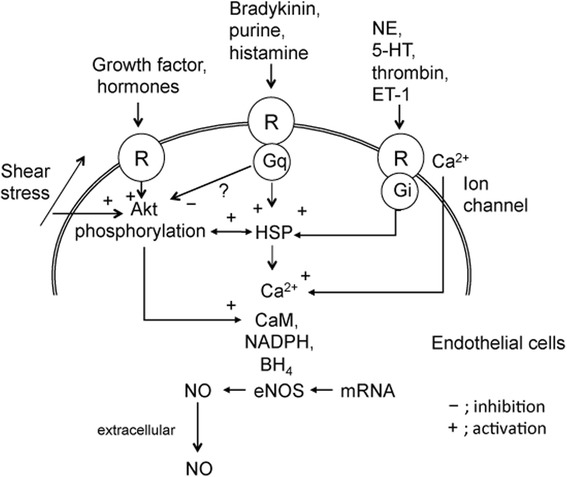
A possible mechanism of nitric oxide production in endothelial cells. Nitric oxide is released through enzymatic conversion of L-arginine by eNOS. eNOS transcription is induced by growth factors and hormones, whereas eNOS enzyme activity requires calcium, calmodulin, NADPH and BH4. The activity of eNOS is regulated by complex formation with these proteins in microdomains of endothelial cells. The L-arginine metabolite, ADMA, reduces production of nitric oxide by competitive binding to eNOS. *Abbreviations:* R, receptor; HSP, heat shock protein; NE; 5-HT, serotonin (5-hydroxytryptamine); ET-1, endothelin-1; CaM, calmodulin; NADPH, nicotinamide adenine dinucleotide phosphate; BH4, 5,6,7,8-tetra-hydrobiopterine; NO, nitric oxide; eNOS, nitric oxide synthase

**Fig. 3:**
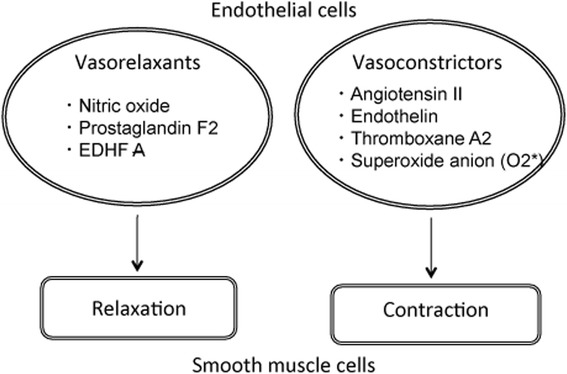
Regulation of relaxation and contraction of smooth muscle cells by vasoactive compounds. Vasoconstriction and vasorelaxation of vascular endothelial cells are controlled by vasoactive molecules such as NO, PGI2, and EDHF that act as vasorelaxants, or vasoconstrictors, which include AII, ET-1, TXA2 and superoxide anion. *Abbreviations:* AII, angiotensin II; ET-1, endothelin; EDHF, endothelium-derived hyperpolarizing factor. NO, nitric oxide; PGI2, prostaglandin F2; TXA2, thromboxane A2. *Abbreviations:* AII, angiotensin II; ET-1, endothelin; EDHF, endothelium-derived hyperpolarizing factor. NO, nitric oxide; PGI2, prostaglandin F2; TXA2, thromboxane A2

Studies in vitro with cultured cells have shown that there are several biomarkers of endothelial dysfunction. Vascular endothelial cell dysfunction is exacerbated when the release of NO is impaired by oxidative stress, ageing, hypertension, and diabetes [39]. In particular, NO plays a key role in the prevention of CAD exerted by endothelial cells. Thus, endothelial cells play important roles in vascular function and in the maintenance of normal homeostasis via the production of biochemical mediators. NO from endothelial cells is generated by eNOS (Fig. 2). Vascular endothelial cells generate vasoactive molecules such as vasorelaxants and vasoconstrictors [51]. NO, PGI2, and EDHF regulate vascular relaxation, and AII and ET-1 stimulate vasoconstriction (Fig. 3).

**Fig. 4:**
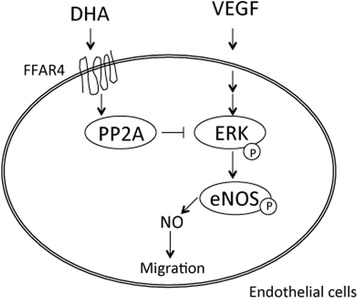
DHA inhibits VEGF-induced endothelial cell migration, which plays a role in angiogenesis and wound repair. The effects of DHA on wound repair and angiogenesis are due, at least in part, to a reduction in cell migration. DHA reduces VEGF-induced cell migration mediated by FFAR4 and the PP2A/ERK1/2/eNOS signaling pathway. *Abbreviations:* DHA, docosahexaenoic acid; FFAR4, Free fatty acid receptor 4; PP2A, protein phosphatase 2A; ERK, extracellular signal-regulated kinase; eNOS, eNOS, endothelial nitric oxide synthase; NO, nitric oxide

The consumption of fish or fish oil influences the expression of various vasoactive molecules and NO production by eNOS gene expression in endothelial cells. Furthermore, DHA induces endothelium-dependent NO-mediated relaxation in the coronary artery. The cells are essential for the transport of metabolites, the control of vascular tone, angiogenesis, and the regulation of hemostasis and blood coagulation [52]. In cultured human coronary artery endothelial cells, DHA enhanced NO production and the activity of eNOS. In addition, it enhanced the expression of eNOS and phospho-eNOS. Specifically, DHA stimulated eNOS and Akt activity, enhanced HSP90 expression, and induced NO bioavailability in response to Akt kinase activation [53]. Earlier studies had demonstrated that DHA prevented TNFα-induced VCAM-1 and monocytic cell adhesion in human endothelial cells [34]. Also, DHA decreased TNF-α-enhanced VCAM-1 expression through inhibition of the NF-κB signaling pathway and AP-1 activation in human aortic endothelial cells [34]. Expression of VCAM-1 and ICAM-1 on the cell surface of human endothelial cells was determined by a cell-surface enzyme-linked immunosorbent assay. In addition, protein expressions of VCAM-1 and ICAM-1 were determined using Western blot analysis [34]. Furthermore, DHA inhibited TNF-α-induced intercellular adhesion molecule 1 (ICAM-1) expression and promoter activity. Synchronously, DHA attenuated TNF-α-induced inhibitory kappa B (IκB) kinase (IKK) phosphorylation and degradation, and p65 nuclear translocation [54]. These effects of DHA were related to Nrf2-mediated HO-1 expression and inhibition of the IKK/NF-κB signaling pathway. Furthermore, DHA inhibited TNF-α-induced phosphorylation of extracellular signal-regulated kinase (ERK), expression of the early growth response gene, ICAM-1, and monocyte cell line HL-60 cell adhesion [55]. DHA may contribute to the inhibition of inflammation induced by TNF-α, and consequently may reduce the risk of atherosclerosis as a consequence of endothelial dysfunction. We recently demonstrated that DHA prevented TNF-α-stimulated gene expression related to dysfunction of human endothelial cells by PCR method, including plasminogen activator inhibitor 1 (PAI-1), LOX-1, and the thromboxane A2 receptor (TXA2R) [33]. It also reversed the TNF-α-mediated decrease of eNOS gene expression. FFAR4 is a G protein-coupled receptor (GPCR) that is activated by n-3 PUFAs such as ALA, EPA, and DHA [35]. With regard to the mode of action of DHA, transfection with small interfering free fatty acid receptors 4 (FFAR4) blocked DHA-mediated inhibition of TNF-α-enhanced ICAM-1 expression and cell adhesion of HL-60. This result indicated that FFAR4 is likely to be associated with the function of DHA. DHA stimulated protein phosphatase 2A (PP2A) enzyme activity and decreased vascular endothelial growth factor (VEGF)-induced phosphorylation of ERK1/2 and eNOS. Moreover, DHA and the FFAR4 agonist GW9508 have been shown to inhibit VEGF-induced cell migration [36]. In this study, human endothelial cells were used as the experimental model, and Western blotting, and assays for MTT, phosphatase activity, and wound-healing were used to explore the effects of DHA on cell migration [36]. The effects of DHA on wound repair and angiogenesis are at least partly owing to a reduction of cell migration. Together, these results indicate that DHA can decrease VEGF-induced cell migration by FFAR4 and via the PP2A/ERK1/2/eNOS signaling pathway [36] (Fig. 4). The activation of FFA4 by FAs induces incretin production in the gut [37] and controls insulin resistance [38] and anti-inflammatory effects in macrophages [56]. These data suggest that stimulation of FFAR4 may enhance metabolic homeostasis.

**Fig. 5:**
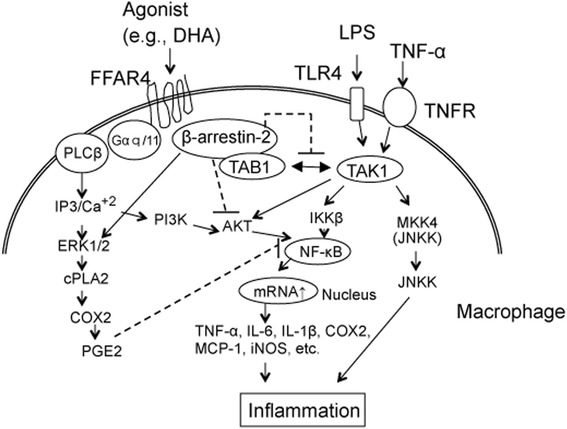
Anti-inflammatory FFA4 signaling pathways in macrophages and mechanisms of action of DHA. Macrophages have FFAR4 signaling pathways that can be affected by anti-inflammatory molecules. LPS and TNF-α induce inflammatory processes following activation of TLR4 and TNFR, which subsequently activate the TAK1 complex of TAK1 and the TAB1 binding protein. Activated TAK1 phosphorylates MKK4 and induces JNK phosphorylation. TAK1 also induces the phosphorylation of IKK-β and NF-κB. NF-κB and phospho-JNK enhance mRNA expression of inflammatory mediators such as TNF-α, IL-6, IL-1β, COX 2, MCP-1 and iNOS. In contrast, FFAR4 together with DHA inhibits TAB1 dissociation and blocks potential TAK1 interactions. Activation of FFAR4 can be induced by β-arrestin-2 enhancement to inhibit its interaction with TAK1. These actions of FFAR4 block downstream activation of NF-κB and JNK to induce anti-inflammatory effects. *Abbreviations:* DHA, docosahexaenoic acid; FFAR4, Free fatty acid receptor 4; ERK 1/2, extracellular signal-regulated kinase 1/2; COX2, cyclooxygenase 2; TAB1, TGF-β activated kinase 1; TAK1, Transforming growth factor beta-activated kinase 1; TLR4, toll-like receptor 4; LPS, lipopolysaccharide; TNFα, tumor necrosis factor-alpha; TNFR, tumor necrosis factor receptor; MKK4; JNKK, Jun-N-terminal kinase kinase; IL-6, interleukin-6; IL-1β, interleukin-1beta; MCP-1, monocyte chemoattractant protein-1; iNOS, inducible nitric oxide synthase

Macrophages also have FFAR4 signaling pathways that can be affected by anti-inflammatory molecules [57] (Fig. 5). A few reports have provided evidence that TAB1 is controlled by FFAR4 stimulation. For example, lipopolysaccharide (LPS) and TNF-α are strong enhancers of inflammatory processes following activation of the toll-like receptor 4 (TLR4) and TNF receptors (TNFRs). In addition, they activate the TAK1 complex of transforming growth factor beta-activated kinase 1 (TAK1) and TGF-β activated kinase 1 (TAB1) binding protein. Activation of either TLR4 or TNFR causes TAK1 to interact with its binding protein TAB1, which activates the TAK1 complex. Activated TAK1 phosphorylates MAPK kinases 4 (MKK4) and enhances the phosphorylation of Jun-N-terminal kinase (JNK). TAK1 also enhances the phosphorylation of IKK-β and NF-κB. NF-κB and phospho-JNK are augmented causing an elevation in the expression of inflammatory mediators such as TNF-α, interleukin-6 (IL-6), IL-1β, cyclooxygenase 2 (COX) 2, monocyte chemoattractant protein-1 (MCP-1), and inducible NOS (iNOS). But FFAR4 inhibits the separation of TAB1 and blocks the potential for interaction with TAK1. On the other hand, activation of FFAR4 can lead to β-arrestin-2 enhancement and inhibit its interaction with TAK1. These actions of FFAR4 inhibit downstream activation of NF-κB and JNK to induce anti-inflammatory effects. These data demonstrate that DHA tends to be more effective in attenuating the effects of compounds in the inflammatory cascade in endothelial cells [58]. FFA4 may also mediate the anti-inflammatory and insulin-sensitizing effects of omega 3 fatty acids [59]. Recently, it was shown that FFAR4 assumed an important role in the efficacy of n-3FA in heart failure [60].

Taken together, DHA affects angiogenesis and wound repair owing to its potential to attenuate cell migration. Furthermore, DHA prevents LPS-induced VCAM-1 and ICAM-1 expression and the adhesion of THP-1 cells to human aortic endothelial cells [61]. DHA inhibited the translocation of TNFR-associated factor 6 (TRAF6) and the phosphorylation of TAK1, p38, and IκBα. These signaling pathways in which DHA can exert effects may be potential targets for the prevention of atherosclerosis.

## Effects of DHA on cardiovascular disease risk factors: Studies in vivo

DHA lowers plasma triglycerides and inflammation, and has been shown to suppress platelet aggregation. Table 2 shows the effects of DHA on risk factors for cardiovascular disease [43, 62].

**Table 2:** Evidence for cardiovascular effects of EPA, DHA and DPA in human sutdies

	EPA	DHA	DPA
Physiologic risk factors			
Lipids	↓ TG levels	↓ TG levels	—
	↓ HDL_3_ cholesterol^2^	↑ LDL particle size	
		↑ HDL_2_ cholesterol	
Vascular and cardiac hemodynamics	Minimal BP effects	↓ BP	—
	? Heart rate effects	↓ Heart rate	
	↑ Cardiac diastolic filling^2^	↑ Cardiac diastolic filling^2^	
	↑ Arterial compliance^2^	↑ Arterial compliance^2^	
Endothelial function	No clear effects^2^	No clear effects^2^	—
Inflammation and oxidative	↓ Inflammation, mixed	↓ Inflammation, mixed	↓ Inflammation, mixed
stress	results^2^	results^2^	results^2,3^
	↓ Oxidative stress, mixed results^2^	↓ Oxidative stress, mixed results^2^	
Thrombosis and coagulation	↓ Collagen-stimulated platelet aggregation^2^	↓ Collagen-stimulated platelet aggregation^2^	↓ Collagen-stimulated platelet aggregation^2^
	Otherwise minimal effects on thrombosis or coagulation	Otherwise minimal effects on thrombosis or coagulation	

1. *BP* blood pressure, *CHD* Coronary heart disease, *DPA* docosapentaenoic, − minimal data available for direct assessment.

2. Base on a single study or few studies. 3. Observation studies only.

*Abbreviations*: *DHA* docosahexaenoic acid, *EPA* eicosapentaenoic acid, *DPA* docosapentaenoic acid, *TG* Triglycerid, *BP* blood pressure, *HDL* high-density lipoprotein, *LDL* low-density lipoprotein, *NO* nitric oxide.

Adapted from reference [43] Mozaffarian and Wu, 2012

### Effect of DHA on plasma triglycerides

As shown in Table 2, a decrease in plasma triglycerides is a common effect of DHA [63]. For example, after fish oil supplementation a decrease in plasma triglycerides and increases in platelet phospholipid arachidonic acid and plasma HDL were observed [64]. Simultaneous increases in plasma HDL, platelet phospholipid EPA, and DHA levels were observed after fish-oil supplementation. Reduced hepatic VLDL synthesis, decreased hepatic enzyme activity for triglyceride synthesis, and enhanced hepatic synthesis of phospholipids contributed to these effects [65, 66].

### Inhibitory effects of DHA on inflammation and CVD

One of the biological effects of n-3 PUFA is to alter the course of inflammatory stimulation. However, in the amounts normally present in the diet it is not known whether DHA reduces inflammation and confers clinical benefit. In several studies, it was shown that supplementation of n-3PUFA decreased the actions of multiple eicosanoids [24, 67]. Furthermore, DHA decreased inflammatory markers such as interleukin-1 beta (IL-1β) and TNFα, IL-6. The potential for the use of DHA to treat diseases associated with inflammation has been demonstrated [68, 69]. During the onset of CVD, the anti-inflammatory effects of n-3 PUFAs may mediate their protective activities against atherosclerosis and plaque rupture [17]. A recent study described effects of ω-3 fatty acids on toll-like receptor 4 and NF-κB p56 in the lung during severe acute pancreatitis in rats [70]. DHA reduced IL-1β-induced VCAM-1, COX2, and VEGFR2 expressions in human intestinal microvascular endothelial cells. Furthermore, it prevented IL-1β-enhanced production of PGE2 and LTB4 in intestinal microvascular endothelial cells. Also, in rats with colitis fish oil rich in EPA and DHA inhibited the production of PGE2 and LTB4 in the colon, and VCAM-1 and VEGFR2 in endothelial cells [71]. It was shown that PUFA exerted these effects by activating a critical modulator of pro-inflammatory cytokines, TLR4 [73]. By contrast, DHA also suppressed inflammation by controlling the expression of COX2 through activation of FFA4 in macrophages [72].

## Preventive effects on CVD of n-3 PUFA and DHA in studies in vivo

CVDs are a group of disorders of the heart and blood vessels, including coronary heart disease, cerebrovascular disease, and peripheral arterial disease. As shown in Table 3, in several meta-analyses of population studies [73–78] and randomised controlled trials [79–85], fish consumption has been shown to prevent coronary heart disease, stroke, and total mortality after stroke in humans [62]. Arteriosclerosis and hypertension are strong risk factors for CVD. Therefore, these effects of DHA could lead to prevention of CVD.

**Table 3:** Studies examining the effect of n-3 fatty acids as fish oils on CHD, stroke and total mortality

Authors	Results of meta-analyses of population studies
Wang et al. (2006) [76]	↓ all-cause mortality; cardiac and sudden death
Bucher et al. (2002) [73]	↓ total mortality; cardiac and sudden death
He et al. (2004) [77]	↓ CHD mortality
Whelton et al. (2004) [75]	↓ fatal and total CHD
He et al. (2004) [77]	↓ stroke, particularly ischaemic stroke
Xun et al. (2012) [78]	↓ stroke, particularly ischaemic stroke
Authors	Results of randomised controlled trials
Burr et al. (1989) [79]	↓ all-cause mortality in post-MI patients
Valagussa et al. (1999) [80]	↓ total and cardiovascular mortality; sudden death in post-MI patients
Tavazzi et al. (2008) [82]	↓ total mortality and hospital admission for CVD in heart failure patients coronary events in patients with hypercholesterolaemia
Yokoyama et al. (2007) (81]	⇄ cardiovascular events in patients at high risk of CVD and impaired glucose or diabetes
Bosch et al. (2012) [83]	⇄ post-operative atrial fibrillation in patients undergoing cardiac surgery
Mozaffarian et al. (2012) [84]	⇄ cardiovascular mortality and morbidity in patients with multiple cardiovascular risk factors

*Abbreviations*: *CHD* coronary heart disease, *CVD* cardiovascular disease.

Adapted from reference [62] Mori et al. 2014

### Arteriosclerosis

Arteriosclerosis is a major cause of heart disorders and cerebrovascular disease. Epidemiologic studies have shown that Japanese men have less atherosclerosis than Caucasian men living in the USA [86]. This study showed that intima-media thickness (IMT) of the carotid artery and coronary artery calcification (CAC) were significantly less in Japanese. Namely, a population-based study in 306 white and 281 Japanese-American men aged 40–49 years was conducted to investigate the IMT of the carotid artery. IMT was associated with the plasma concentration of n-3 PUFAs in Japanese men, but a similar association was not observed for coronary artery disease (CAD). Previous reports had found that an extremely high intake of n-3 PUFAs inhibits atherosclerosis [87]. On the other hand, another report showed that DHA prevented chronic intermittent hypoxia-induced atherosclerosis but did not improve atherosclerosis in control apolipoprotein-E deficient mice [88]. In the aorta, expression of matrix metalloproteinase-2 (MMP-2) was found to be decreased by DHA. In 160 Japanese patients with CAD [54] the serum level of DHA was related to endothelial function as evaluated by flow-mediated dilation (FMD) in patients with CAD. These results suggest that a low serum level of DHA may be a biomarker of endothelial dysfunction, and that DHA may prevent chronic intermittent hypoxia-induced atherosclerosis [89].

### Hypertension

The active components in fish oil that underlie its antihypertensive effect are thought to be EPA and DHA. DHA and EPA have been shown to decrease BP in subjects with essential hypertension [90]. Namely, in men and women with mild essential hypertension (*n* = 156), the effects of 6 g per day of 85% EPA and DHA were examined in a 10-week dietary study. EPA and DHA decreased the BP in essential hypertension [92]. An international cross-sectional epidemiologic study of 4680 subjects in China, Japan, UK, and USA found that dietary n-3 LC-PUFAs were inversely associated with BP [91]. The results of this study indicate that foods containing n-3 PUFA may contribute to the prevention and control of adverse BP levels. Furthermore, in a recent intervention study it was determined that dietary intakes of DHA and EPA significantly reduced BP [92]. Results from 70 randomized controlled trials demonstrated that consumption of ≧ 2 g EPA daily plus DHA reduces systolic and diastolic BP. DHA reduced ambulatory systolic and diastolic BP and heart rate (HR) in 56 mildly hyperlipidemic obese men [93]. In the study design, 59 subjects were randomly assigned to consume 4 g EPA, DHA, or olive oil/day for 6 weeks. DHA intake reduced systolic and diastolic BP and heart rate (HR), whereas EPA had no significant effect on either BP or HR. These results suggested that DHA, but not EPA, may decrease BP and HR in men. In a more recent meta-analysis of randomized controlled trials, EPA plus DHA was found to reduce both systolic and diastolic BP relative to a placebo [92]. BP was reduced by EPA plus DHA (2 g daily) in both normotensive and hypertensive subjects, and the effect was particularly strong in those with untreated hypertension. Lower doses of EPA plus DHA (between 1 and 2 g/day) might reduce systolic BP but not diastolic BP. In another study, the effects of DHA and EPA were investigated in relation to BP, serum lipids, and glycemic control in patients with type 2 diabetes [94]. Study design were 59 subjects were randomly assigned to consume 4 g EPA, DHA, or olive oil/day for 6 weeks. DHA and EPA had similar beneficial effects on lipids, but inverse effects on short-term glycemic management and BP control in hypertensive type 2 diabetics. This result indicated that the effect of DHA on hypertension necessitates long-term evaluation. Furthermore, the lowering effects of DHA on BP, as well as its effects on endothelial and cardiac function, were confirmed in meta-analyses. DHA may influence both BP and heart conditions, as assessed by HR and electrocardiogram (ECG), and may control adrenergic function [95].

In spontaneously hypertensive rats (SHR), the anti-hypertensive effect of intake of DHA, EPA, and gamma-Linolenic acid (GLA) was compared with that in normotensive Wistar Kyoto rats (WKY) [96]. SHR rats and WKY rats were fed for 10 weeks a diet containing either 5.65 g/kg GLA, 6.39 g/kg EPA, or 4.94 g/kg DHA. In SHR, dietary supplementation with DHA significantly decreased BP, enhanced total antioxidant status, and reduced the speed of platelet aggregation. These results indicated that DHA not only reduces BP but also improves oxidation status and reduces platelet aggregation in hypertensive rats.

A recent randomized controlled trial indicated that daily doses of EPA plus DHA as low as 0.7 g achieve clinically useful BP reductions, and might be associated with lower CVD risk [97]. In this double-blind placebo-controlled RCT, normal men and women (*n* = 312) were given fish oil containing EPA plus 0.7 or 1.8 g of DHA per day for eight weeks.

### Coronary artery disease

Many epidemiologic studies have observed an inverse association between n-3 PUFA intake and CVD. A high intake of n-3 PUFAs in fish or fish oil supplements reduced all-cause mortality, including mortality from heart disease and sudden death [76]. Several studies have recorded an inverse relation between n-3 LC-PUFAs and coronary heart disease (CHD) [73, 98, 99]. A meta-analysis of seventeen cohort studies conducted by [99] searched the databases of PubMed and the Institute of Science Index from to 2010. Data from relevant studies were retrieved from 315,812 participants collected over nine to 15 years. In the pooled data, a dose-response analysis showed that every additional 15 g/day of fish intake decreased the risk of CHD mortality by 6%. This study also demonstrated that both low and moderate fish intake decrease CHD mortality. Furthermore, in 517 patients in urban Japan who were on maintenance hemodialysis, alterations in their serum PUFA profiles during therapy were associated with CVD risk [100].

The blood levels of n-3 PUFAs can vary depending on a person’s location and dietary habits, and these differences may influence the preventive effects conferred by DHA. These reports and other studies concluded that enhanced seafood intake is beneficial and may contribute to the prevention of CVD [83–85]. Other recent research in the patients with hypertriglyceridemia and low HDL cholesterol (HDL-C) showed that the combination of a statin and n-3 FFA was effective for the treatment of coronary artery disease [101].

The effects of DHA on endothelial cell function in patients with CAD have been investigated. The results demonstrated a relation between the circulating concentration of DHA and CAD in Japanese patients; serum DHA was also associated with endothelial function as evaluated by FMD [96]. Evidence that DHA may inhibit disorders of endothelial functional induced by high-fat diets was obtained from studies in 20 healthy humans by Fahs et al. (2010) [102]. Namely, brachial artery FMD, forearm blood flow (FBF), total hyperemia, central and peripheral BP, and central artery stiffness were investigated in 10 healthy men and 10 healthy women after a high-fat diet supplemented with either a placebo or approximately 1 g EPA and DHA. On the other hand, treatment with DHA (2 g/day) enhanced modified brachial artery blood flow and conductance during exercise in humans. Thus, high concentrations of DHA may contribute to the prevention of endothelial dysfunction. These effects of DHA may mitigate CVD risk and exercise intolerance due to heart failure by preventing endothelial dysfunction [103].

### Cerebrovascular disease (stroke)

Cerebrovascular disease, in particular stroke, is a severe life-threatening condition that occurs when the blood flow to part of the brain is interrupted. Several studies have investigated whether neuroprotection and long-term survival are enhanced by fish oil and DHA. Transient global cerebral ischemia-induced retrograde amnesia was reversed by fish oil [104], indicating that intake of fish oil ameliorates cerebral infarction. Fish oil might be therapeutic to neural dysfunction associated with learning and memory defects after cerebral ischemia. In addition, DHA had a protective effect on neurological disorders, such as neuronal cell death induced by middle cerebral artery occlusion (MCAO), in Sprague-Dawley (SD) rats [105]. DHA treatment reduced tissue loss and total and cortical infarct volumes compared to those in a saline-treated group. With regard to the mechanism, the inflammatory reaction of cells plays an important role in cerebral ischemic brain injury, and anti-inflammatory treatment of stroke is beneficial. In other words, dietary DHA has anti-inflammatory and neuroprotective effects against ischemic stroke. For example, in a rat model of permanent cerebral ischemia, DHA induced a neuroprotective effect against ischemia by decreasing brain infarction, edema, and disruption of the blood-brain barrier [106]. In this study, the results of Western blot, enzymatic assay, quantitative PCR, and flow cytometric analysis revealed that DHA decreased microglia activation, leukocyte infiltration, pro-inflammatory cytokine induction, and leukocyte activation after cerebral ischemia. Namely, DHA inhibited anti-inflammatory events, such as macrophages/microglia activation, leukocyte infiltration, pro-inflammatory cytokine expression, and leukocyte activation after cerebral ischemia.

A low serum n-3 PUFA/n-6 PUFA ratio predicted neurological deterioration in Japanese patients with acute ischemic stroke [107]. Furthermore, disease of cerebral small vessel (SVDs) is associated with stroke or diminished cognitive dysfunction. A study of 220 patients with cerebral infarction indicated that reduction of the percentage EPA, DHA, and n-3-PUFAs was related to the presence of cerebral micro-bleeds, high-grade white matter changes, and high-grade perivascular spaces, but not asymptomatic lacunar infarctions [108]. In addition, SVD scores were negatively correlated with the proportion of EPA, DHA and n-3-PUFAs. These results suggest that reduction of the level of n-3-PUFA in blood may induce small vessel disease in the brain in acute ischemic stroke patients.

DHA has also been found to protect neuronal cells after global cerebral ischemia reperfusion. In a rat vascular occlusion model, DHA inhibited hippocampal neuronal cell death [109] by decreasing Bax expression and increasing Bcl2 expression. These results suggest that DHA prevented ischemic stress-induced neuronal cell death. DHA has several other functions besides neuroprotection in cerebrovascular disorders by way of effects on neuronal excitation [110], memory [111], and photoreceptors [112, 114]. For example, there is evidence that it increases neuroprotectin D1 (NPD1) and may prevent cerebrovascular disease including ischemic stroke [113, 114].

## Conclusion

In this review, the relation between the protective effects of fish oil fatty acids such as DHA and CVD is discussed. Many studies have shown that DHA has an important role in the function of vascular endothelial cells. During regular consumption of n-3 PUFAs, DHA has the potential to decrease endothelial dysfunction and prevent CVD through effects on endothelial metabolism, inflammation, thrombosis, and arrhythmia. Thus, dietary DHA prevents endothelial dysfunction and regulates vascular health. Intake of foods rich in DHA such as fish has the potential to make a significant contribution to the prevention of CVD.

## Abbreviations

ACE: angiotensin converting enzyme;
ALA: Alpha-linolenic acid
BP: blood pressure
CVD: cardiovascular disease
DHA: docosahexaenoic acid
EDHF: endothelium-derived hyperpolarizing factor
eNOS: nitric oxide synthase
EPA: eicosapentaenoic acid
ET-1: endothelin-1
FFAR4: Free fatty acid receptor 4
HDL: high-density lipoprotein
ICAM-1: intercellular adhesion molecule 1
LDL: low-density lipoprotein
LOX-1: oxidized low density lipoprotein receptor 1
LPS: lipopolysaccharide
n-3 FA: omega-3 fatty acids
NPD1: neuroprotectin D1
PAI-1: plasminogen activator inhibitor 1
PUFAs: polyunsaturated fatty acids
TLR4: toll-like receptor 4
TXA2R: thromboxane A2 receptor
VCAM-1: vascular cell adhesion molecule-1
